# Impact of baseline telomere length on survival and chemotherapy related toxicity in breast cancer patients receiving (neo)adjuvant anthracycline containing chemotherapy

**DOI:** 10.1016/j.tranon.2022.101551

**Published:** 2022-10-08

**Authors:** Sigrid Hatse, Marta Serena, Christof Vulsteke, Kevin Punie, Patrick Neven, Ann Smeets, Annouschka Laenen, Hans Wildiers

**Affiliations:** a Department of Oncology, Laboratory of Experimental Oncology, KU Leuven, Leuven, Belgium; b Department of General Medical Oncology, Leuven Cancer Institute, University Hospitals Leuven, Leuven, Belgium; c Multidisciplinary Breast Centre, Leuven Cancer Institute, University Hospitals Leuven, Leuven, Belgium; d Interuniversity Centre for Biostatistics and Statistical Bioinformatics, Leuven, Belgium

**Keywords:** Breast cancer, Telomere length, Prognosis, Outcome, Toxicity, TL, telomere length, FEC, fluorouracil, epirubicin and cyclophosphamide, D, docetaxel, DDFS, Distant disease-free survival, BCSS, breast cancer-specific survival, OS, overall survival, BC, breast cancer, G-CSF, granulocyte colony-stimulating factor, RDI, relative dose intensity, BMI, body mass index, FN, febrile neutropenia, LVEF, left ventricular ejection fraction

## Abstract

**Purpose:**

The aim of this study is to assess baseline mean leukocyte telomere length (TL) as a potential predictive factor for chemotherapy toxicity and a prognostic marker for long-term outcome in early breast cancer (BC) patients.

**Methods:**

445 BC patients were selected, diagnosed between 2007 and 2010 with early BC and treated with (neo)adjuvant fluorouracil, epirubicin and cyclophosphamide (FEC) or with FEC and Docetaxel (FEC-D). RT-qPCR was performed on germline DNA samples collected at diagnosis before any treatment, to measure mean leukocyte TL. Uni- and multivariable logistic regression or Cox proportional hazard regression analyses were carried out to assess correlation between baseline TL and toxicity parameters (derived from the medical chart) or longer-term outcome.

**Results:**

Baseline TL correlated with age as expected (*p* = 0.005), but not with febrile neutropenia (*n* = 97), left ventricular ejection fraction >10% decrease (*n* = 17) nor other toxicity endpoints measured (all *p* > 0.05). TL was neither associated with overall survival, breast cancer specific survival or distant disease-free survival (all *p* > 0.05).

**Conclusions:**

Baseline TL is not associated with chemotherapy-related toxicity nor long-term outcome in BC patients.

## Highlights

• Telomere length correlates with chronological age.
• This study evaluates telomere length in patients diagnosed with early breast cancer.
• Surprisingly, telomere length did not predict chemotherapy related toxicity.
• In addition, baseline telomere length is not associated with relapse and survival.

## Introduction

Breast cancer (BC) is the most frequently diagnosed cancer and still remains the leading cause of cancer death among females worldwide [1]. (Neo)adjuvant chemotherapy can improve outcome significantly, but has been associated with both short and long term side effects [2], [3], [4], [5], [6]. Acute or short-term toxicities include hematological toxicity (neutropenia, thrombocytopenia, anemia, ...), infection, nausea and vomiting, ... [7]. These side effects usually resolve when the treatment is completed. However, in some cases they can lead to dose reduction and thus reduced RDI (relative dose intensity), which is the ratio of delivered dose intensity of chemotherapy to standard dose intensity. Obviously, these changes on treatment may have a negative impact on the patient survival [8]. On the other hand, late or long-term toxicities can appear or persist until years after the primary treatment, and include cardiac/cardiovascular disease, neuropathy, cognitive dysfunction, ovarian failure, premature menopause and second tumors (mostly leukemia). Chemotherapy-related toxicities vary among the different chemotherapeutic regimens [5,6]. For example, neuropathy is strongly associated with the use of taxanes, while cardiac toxicity has been related to anthracyclines. Understanding the relationship between patients’ characteristics and these symptoms can help to identify patients who are at higher risk of developing chemotherapy-related toxicities. This would allow to individualize decisions regarding benefit-to-risk ratio and, if needed, application of prophylactic measures such as G-CSF (granulocyte colony-stimulating factors), dose modification or alternative chemotherapy regimens [9]. With this aim, previous studies have analyzed several potential predictive factors such as age (haematological toxicity) or prior cardiac disease (cardiac dysfunction). However, they have reported inconsistent and imperfect results [9], [10], [11], [12], [13], [14], and more solid predictive factors for chemotherapy induced severe side effects are eagerly awaited.

Telomeres are noncoding sequences that stabilize chromosome ends and prevent them from degradation and end-to-end fusion [15]. They are composed of double-stranded tandem repeats of 5’-TTAGGG-3’ that shorten by each round of cell division because of incomplete end replication. Telomere shortening contributes to the mitotic clock: when telomeres (or even one single telomere within the cell) become critically short, the cell reaches the so-called ‘Hayflick limit’ and enters a state of replicative senescence, implying that it remains metabolically active but it can no longer divide [16]. Thus, telomere length (TL) is commonly used as an indicator of biological age. Although a negative correlation between telomere length and chronological age clearly exists [17], the rate of telomere attrition – and thus biological aging – strongly varies among individuals and is influenced by many (lifestyle-related) factors, such as stress, smoking, physical exercise. In cancer patients, intensive hematopoietic stem cell proliferation, required for hematologic repopulation after each chemotherapy cycle, may obviously speed up telomere attrition and hence, it could be hypothesized that chemotherapy accelerates the aging process [18,19]. Also, chemotherapy-induced telomere shortening is expected to exert differential impact in a younger population (despite TL shortening, no telomere crisis is expected to appear) compared to an older one. Patients who already have short telomeres might be more susceptible to chemotherapy-induced (hematological) toxicity [20,21]. We aimed to test the prognostic value of TL for chemotherapy induced toxicity in a large early breast cancer population treated with an anthracycline and/or taxane regimen.

In addition, TL might also be useful as a predictive factor for cancer outcome, possibly related to decreased RDI, but also related to potentially altered susceptibility of ‘older’ (shorter TL) tumor cells to chemotherapy. A second aim of our study was thus to investigate the relation between TL and longer-term outcome in this breast cancer population.

## Methods

### Study population

Since 2000, breast cancer patients visiting the Leuven Multidisciplinary Breast Cancer Center have systematically been entered into a clinical database, containing patient and tumor-related information, as well as clinical follow-up such as treatment, relapse and cause of death. Since 2007, germline (leukocyte) DNA is prospectively collected from all consenting patients at baseline before any therapy, and stored at –80 °C. We previously published on the impact of SNPs in 980 early breast cancer patients on chemotherapy induced toxicity and outcome [22]. In nearly half of these patients, germline DNA was collected after initial breast cancer treatment. For the present study, we selected 445 patients from this cohort, diagnosed with non-metastatic invasive breast cancer between June 2007 and October 2010, with a baseline germline DNA sample available, and who had received (neo)adjuvant fluorouracil, epirubicin and cyclophosphamide (FEC) or FEC-Docetaxel (FEC-D) chemotherapy which were the standard (neo)adjuvant chemotherapy regimens in that period (docetaxel was reimbursed only for node positive breast cancer in Belgium during the majority of the study period). Primary G-CSF use was only reimbursed for patients above age 65. If no primary G-CSF was given, a nadir count was done at day 10 and day 15 of the first cycle. In case of prolonged grade III neutropenia (≥ 5 days), secondary prophylaxis with G-CSF was given for the next cycles of chemotherapy. In case of febrile neutropenia, secondary prophylaxis with G-CSF was also recommended for the next cycles. In the FEC only arms (*n* = 132), FEC was generally planned for 6 cycles, except for 31 patients who were preplanned to receive 3 cycles and one patient who was preplanned to receive 4 cycles. FEC-docetaxel (*n* = 313) consisted of 3 cycles of FEC, followed by 3 cycles of docetaxel. Both regimens were given according to the French PACS-01 study [23].

All patients were staged by TNM as defined by The American Joint Committee on Cancer staging system (8th edition) [24]. Surrogate subtype was defined based on clinicopathological aspects according to the St. Gallen guidelines as described in Brouckaert et al. [25].

All patients included in the study gave written informed consent for blood sampling and biomarker research at first diagnosis of breast cancer. Blood sampling, collection of patient data and genetic analysis were approved by the local ethics committee (University Hospitals Leuven).

### Outcome parameters

Distant disease-free survival (DDFS), breast cancer-specific survival (BCSS) and overall survival (OS) were defined according to the Guidelines for time-to-event end point definitions in breast cancer trials, provided by the DATECAN initiative (Definition for the Assessment of Time-to-event Endpoint in Cancer trials) [26]. Toxicity parameters were defined according to Common Terminology Criteria for Adverse Events (CTCAE) 3.0 [27].

The following toxicity events were retrospectively retrieved from the medical chart as described previously [28]: (i) hematological toxicities including febrile neutropenia (FN), prolonged grade 4 neutropenia (≥5 days), deep neutropenia (<100/μl), grade 3 bleeding, grade 3–4 thrombocytopenia and grade 3–4 anemia, (ii) non-hematological toxicities including diarrhea, mucositis and (iii) cardiac toxicity. Cardiac toxicity was described as a cardiac failure grade 3–5 or a LVEF decrease >10% from baseline. We focused on these main toxicities as we expected that other serious side effects would be rare not allowing solid statistical conclusions. Neuropathy was not included since not all patients received docetaxel and those who did, only received 3 cycles of docetaxel, which is less than the currently used 6 cycles in the popular regimen ‘docetaxel cyclophosphamide’. Neuropathy is likely to be much less an issue with 3 *versus* 6 cycles. Moreover, docetaxel is associated with less severe neurotoxicity compared to paclitaxel. For these reasons, our cohort did not seem appropriate for an analysis focusing on neuropathy. Outcome information including last date of follow-up, development of distant metastases, death and cause of death was also retrieved. The majority of patients treated at the University Hospitals Leuven are followed on the long term (>10y) every 2y or even yearly. The electronic record (used since before 2000) easily allows survival analyses. Moreover, since the last years, the Belgian health authorities established a ‘national’ medical file allowing survival follow-up even if patients were followed outside our hospital. Therefore, the amount of missing or uncaptured long-term information was limited.

### Blood sampling

Peripheral blood was sampled in 4 mL BD Vacutainer EDTA K2E tubes and blood samples were processed within 60 min. After centrifugation at 1600xg for 10 min at 4°C, the supernatant (plasma) was isolated and the precipitated cell fraction of the EDTA tube was stored at -20°C, until germline DNA extraction was performed using the QIAamp DNA Blood Midi kit (QIAGEN, cat.nr. 51185), following the manufacturer's instructions. DNA concentration was measured by NanoDrop 2000/2000c.

### Mean leukocyte telomere length (T/S ratio)

Mean leukocyte telomere length was assessed by qPCR [29]. In this method, the relative amount of telomeric DNA (T/S ratio) is calculated based on the Cp values obtained for telomeric DNA (T) and for the single-copy housekeeping gene 36B4 (S), measured in the same sample. To this end, two separate real time quantitative SYBR Green-based PCRs were performed in parallel for each sample, with the telomeres (TEL) and the single copy gene (36B4) as the respective target genes. All samples were assayed in triplicate wells. Each run included a series of standards (i.e. 100, 25, 6.25 and 1.56 ng/well, also in triplicate) prepared from commercially available reference DNA (Human Genomic DNA, Roche cat. no. 11691112001), as well as negative controls without template DNA.

Primer pairs used were 5’-ACACTAAGGTTTGGGTTTGGGTTTGGGTTTGGGTTAGTGT-3’ and 5’-TGTTAGGTATCCCTATCCCTATCCCTATCCCTATCCCTAACA-3’ for telomeres and 5’-CAGCAAGTGGGAAGGTGTAATCC-3’ and 5’-CCCATTCTATCATCAACGGGTACAA-3’ for 36B4. The reaction mixture (total volume of 20 µL) included: 1x LightCycler 480 SYBR Green I Master (Roche); either telomere primers at 0.6 µM each or 36B4 primers at 0.5 µM each; 16 ng of template DNA (for sample wells) or the appropriate amount of reference DNA (for standard wells). Plates were run on a Roche LightCycler 480 platform, using the following thermal cycling program (identical for both telomeres and 36B4): activation for 10 min at 95°C; two initiation cycles of 15s at 95°C followed by 15s at 49°C; 35 amplification cycles of 15s at 95°C, 10s at 60°C and 15s at 72°C. Finally, melting curves were established in order to check amplicon purity (Tm = 79.2°C for telomere PCR and 81.1°C for 36B4 PCR).

The T/S ratio for an experimental sample is the amount (ng) of standard DNA that matches the experimental sample for Cp value of the telomere PCR (T), divided by the amount (ng) of standard DNA that matches the experimental sample for Cp value of the single-copy gene 36B4 (S). Samples with a T/S >1 (<1) have an average TL greater (shorter) than that of the standard DNA [29,30].

### Statistical analysis

Kaplan-Meier estimates were used for presenting overall survival. The cumulative incidence function was used for breast cancer-specific survival and distant disease-free survival. Cox models were used to analyze the effect of TL on survival outcomes, and logistic regression was used for binary toxicity outcomes. Results are presented for univariable analyses and for multivariable analysis, correcting for known possible confounders. Acute toxicity endpoints (hematological, diarrhea and mucositis) were adjusted for age, BMI, chemotherapy regimen and use of growth factors. Correction for age was incorporated in the multivariable model, since the purpose of our study was to assess TL by itself, as a marker of biological age, for its predictive capacity with regard to chemotherapy toxicity and outcome, apart from chronological age. Long term outcome endpoints (survival and cardiac toxicity) were adjusted for age, BMI, tumor stage and subtype, chemotherapy regimen, received cycles, global RDI and use of growth factors (G-CSF: none, primary, secondary). The number of cycles and RDI were not integrated in acute toxicity because of potential interference, whereas they were included for long term endpoints, since there may be a causal relation between both.

The Mann-Whitney U test was used to assess differences in TL between the groups. The Spearman correlation coefficient was used to quantify the association between TL and age. Analyses have been performed using SAS software (version 9.4 of the SAS System for Windows).

## Results

### Description of the cohort

Patient and clinical characteristics of the study group are shown in Table 1. Mean age at BC diagnosis was 50.9 ± 9.9 years and mean BMI was 24.8 ± 4.7. Tumor subtype and stage are also shown, and reflect a population where (neo)adjuvant chemotherapy is used.

**Table 1:** Study population characteristics.

**Patient characteristics**	**Mean ± SD or frequency (%)**
Number of patients (N)	445
Age at diagnosis (years)	50.9 ± 9.9
Body mass index (kg/m2)	24.8 ± 4.7
**Tumor stage**	
I	45 (10.1%)
IIA	147 (33.0%)
IIB	114 (25.3%)
IIIA	70 (15.7%)
IIIB	23 (5.2%)
IIIC	46 (10.3%)
**Tumor subtype**	
Luminal A like	134 (30.1%)
Luminal B like (HER2 negative)	128 (28.8%)
Luminal B like (HER2 positive)	49 (11.0%)
HER2+	28 (6.3%)
Triple negative	106 (23.8%)
**Chemotherapy regimen**	
FEC	132 (29.7%)
FEC-docetaxel	313 (70.3%)
**Received cycles (sum)**	
1	4 (0.9%)
2	3 (0.7%)
3	31 (7.0%)
4	8 (1.8%)
5	4 (0.9%)
6	393 (88.3%)
7	2 (0.5%)
**Global RDI (relative dose intensity)**	
≤0.95	80 (18.0%)
>0.95	393 (88.3%)
**Growth factor use**	
None	216 (48.5%)
Primary prophylaxis	125 (28.1%)
Secondary prophylaxis	104 (23.4%)

Of the 445 eligible patients, 132 (29.7%) were treated with FEC only, of whom 122 completed the planned treatment (3–6 cycles received); the other 313 (70.3%) were treated with FEC-T, of whom 304 completed the planned treatment (3 cycles received of each). In total, 393 (88.3%) patients received 6 cycles of chemotherapy. The global RDI was >0.95 for 365 (82.0%) patients, whereas significant dose reduction or omission (RDI ≤ 0.95) had to be applied for 80 (18.0%) patients. Primary prophylactic G-CSF (before any FN event occurred) was given to 125 patients (28.1%), while the majority received no G-CSF (*N* = 216; 48.5%) (Table 1).

Within the entire cohort, the median T/S ratio measured on leukocyte DNA was 0.98 (IQR=[0.80;1.33]).

As shown in Table 2, the most common hematological toxicity was prolonged grade IV neutropenia, which was observed in 51.9% of patients. Febrile neutropenia in any cycle occurred in 97 (21.8%) patients, of whom 69 (15.5%) had early FN (in the first cycle). Deep neutropenia also occurred in 69 patients (15.5%). Other hematological toxicities such as grade 3 bleeding, grade 3–4 thrombocytopenia and grade 3–4 anemia, were rare (<2.5% of patients), as well as non-hematological toxicities (diarrhea, mucositis, neuropathy). Cardiac toxicity data were only available for 364 patients. Cardiac failure was only observed in 1 patient, and LVEF >10% decline in 17 cases (Table 2).

**Table 2:** Toxicity occurrence in the study population.

**Toxicity event**	**Frequency (%)**
**Hematological (***n* = **445)**	
Febrile neutropenia	97 (21.8%)
Early FN (first cycle)	69 (15.5%)
Late FN (after first cycle)	28 (6.3%)
Prolonged grade IV neutropenia (>= 5 days)	231 (51.9%)
Deep neutropenia (<100/µl)	69 (15.5%)
Grade 3 bleeding	1 (0.2%)
Grade 3–4 thrombocytopenia	1 (0.2%)
Grade 3–4 anemia	10 (2.3%)
**Non-hematological (***n* = **445)**	
Diarrhea grade 3–4	4 (0.9%)
Mucositis grade 3–4	5 (1.1%)
**Cardiac toxicity (***n* = **364)**	
Cardiac failure grade 3–5	1 (0.3%)
LVEF decrease >10% from baseline	17 (4.7%)

### Survival analysis

Outcome variables are summarized in Table 3. During the follow up (median of 8.6 years, IQR = [8.0; 9.4]), 30 patients had a local relapse (6.7%), 70 developed distant metastasis (15.7%) and 63 died (14.2%).

**Table 3:** Outcome variables of the study population.

**Outcome variables**	**Frequency (%)**
**Relapse (***n* = **445)**	
Local relapse	30 (6.7%)
Distant metastasis	70 (15.7%)
**Survival (***n* = **445)**	
Total deaths	63 (14.2%)
Death from breast cancer	50 (11.2%)
Death from other cause	6 (1.4%)
Death from unknown cause	7 (1.6%)

OS was 97.7% (95% CI [95.8–98.8]) at 2 years, 92.1% (95% CI [89.1–94.2]) at 5 years and 81.2% (95% CI [72.5–87.4]) at 10 years. For 56 of the 63 patients who died during the follow-up, the cause of death was documented: 50 died from breast cancer, while 6 died from other causes. The BCSS estimates were 97.9% (95% CI [96.3–99.0]) at 2 years, 92.4% (95% CI [89.7–94.7]) at 5 years and 86.5% (95% CI [81.8–90.5]) at 10 years. For the remaining 7 patients, the cause of death was unknown.

Of the 445 patients, 70 (15.7%) developed distant metastasis, of whom 50 eventually died from breast cancer, 2 died from unknown causes and 18 were still alive at last follow-up. The DDFS estimates were 93.7% (91.1–95.7) at 2 years, 88.2% (85.0–91.0) at 5 years and 83.0% (79.1–86.6) at 10 years.

### Association between TL and age

As expected, there was a weak but significant inverse correlation between age and telomere length: the Spearman correlation coefficient was -0.133 with a 95% CI of [-0.223; -0.040] and a *p*-value of 0.005. These results confirm the well-established negative association between TL and age, older age being associated with shorter TL.

### Association between TL and toxicities

In univariable analysis, we found that a longer TL (higher T/S ratio) was associated with a lower risk of prolonged grade IV neutropenia (Table 4). However, after correcting for potential confounding factors (age, BMI, chemotherapy regimen and use of G-CSF), there was no evidence for an association between TL at diagnosis and any type of neutropenia (all *p*-values > 0.05).

**Table 4:** Impact of telomere length on hematological and cardiac toxicities using uni- and multivariable analysis.

**Type of toxicity**	**Correction**^a^	**Odds Ratio (95% CI)**	*p*-value	**N**	**events**
Early FN	No	0.81 (0.58;1.12)	0.1955	445	69
Early FN	Yes	0.80 (0.56;1.13)	0.1964	445	69
Early or late FN	No	0.77 (0.58;1.04)	0.0883	445	97
Early or late FN	Yes	0.78 (0.57;1.06)	0.1057	445	97
Prolonged grade IV neutropenia	No	0.83 (0.69;1.00)	**0.0458**	445	231
Prolonged grade IV neutropenia	Yes	0.84 (0.68;1.04)	0.1060	445	231
Deep neutropenia	No	0.89 (0.68;1.16)	0.3882	445	69
Deep neutropenia	Yes	0.91 (0.69;1.19)	0.4796	445	69
LVEF decrease >10%	No	0.91 (0.56;1.47)	0.1955	364	17
LVEF decrease >10%	Yes	0.84 (0.49;1.45)	0.1964	364	17

CI: confidence interval; Odds ratio (OR) given for 1-unit increase in T/S ratio

OR >(<)1 means higher (lower) risk for increasing T/S ratio

a Hematological toxicities correction for: age, BMI, chemotherapy regimen and use of growth factors (primary/other); LVEF decrease >10% correction for: age, BMI, received cycles of FEC and global RDI

Grade 3 bleeding and grade 3–4 thrombocytopenia could not be further analyzed because only one event occurred for each. Likewise, the low number of events for grade 3–4 anemia, diarrhea and mucositis did not allow multivariable analysis, but exploratory univariable analysis did not reveal any significant association with TL (all *p* > 0,05, data not shown).

Concerning cardiac toxicity, there was only one case with cardiac failure grade 3–5, and 17 with a LVEF decrease >10%. Multivariable analysis of LVEF decrease >10% adjusting for possible confounders (age, BMI, received cycles of FEC and RDI) did not show a significant association between TL at diagnosis and cardiac toxicity (*p* > 0.05) (Table 4).

### Association between TL and outcome

Cox models were used to analyze the effect of TL on survival outcomes. Results from univariable analysis (no correction) and multivariable analysis (correcting for age, BMI, tumor stage and subtype, chemotherapy regimen, received cycles, global RDI and use of growth factors) are presented in Table 5. All *p*-values are > 0.05; we can thus conclude that there is no evidence for an association between TL measured at diagnosis and OS, BCSS nor DDFS.

**Table 5:** Uni- and multivariable analysis of association of TL with OS, BCSS and DDFS.

**Outcome**	**Correction**^a^	**Hazard Ratio (95% CI)**	*p*-value	**N**	**events**
OS	No	1.07 (0.948;1.217)	0.2838	445	62
OS	Yes	1.07 (0.947;1.232)	0.2789	445	62
BCSS	No	1.09 (0.961;1.232)	0.1813	439	50
BCSS	Yes	1.08 (0.956;1.229)	0.2066	439	50
DDFS	No	1.08 (0.965;1.209)	0.1776	445	70
DDFS	Yes	1.06 (0.953;1.186)	0.2712	445	70

CI: confidence interval; Hazard Ratio (HR) given for 1-unit increase in T/S ratio; HR >(<)1 means higher (lower) risk for increasing T/S ratio

a Correction for: age, BMI, stage, subtype, chemotherapy regimen, received cycles, global RDI and use of growth factors (none, primary, secondary).

## Discussion

In this study, we examined the possibility that TL could serve as a predictive factor to identify those patients with higher risk of developing chemotherapy-related toxicities. It has been previously shown that TL is an indicator of the proliferative capacity of the cells, which is tightly linked to their ability to renovate tissues in stressful situations, for instance during and after chemotherapeutic treatment [31]. Given this, we hypothesized that patients with shorter TL would have lower (hematopoietic) renewal capacity, and would therefore develop more severe and/or frequent chemotherapy-related toxicities, which are caused by the effect of the drugs on healthy (non-cancer) cells. However, T/S ratio did not correlate with any type of toxicity in our cohort. A few other studies have been published concerning TL and chemotherapy-related side effects. Our results are in conformity with a study which did not find any significant correlation between TL and side effects in bladder cancer patients receiving aMVAC (accelerated methotrexate, vinblastine, doxorubicin and cisplatin) [32]. However, rate of side effects was also low in this study, at least for some of the toxicities under analysis. In contrast, one study significantly correlated shorter leukocyte TL with higher risk of non-hematological toxicity in elderly ovarian cancer patients, but did not find such association for hematological events [33]. The cohort of this study received carboplatin instead of FEC or FEC-D, so it is possible that the observed correlation is drug-specific. Moreover, it is not surprising that correlations of chemotherapy-induced adverse events with a well-established aging biomarker such as TL, are more prominent in an elderly population, where both toxicity events and short telomeres are more frequent than in our current cohort, which is relatively young (mean age 50). The fact that our study population is younger than the median age of breast cancer (>60y) indicates that the FEC and FEC-D regimens are considered relatively toxic, and not feasible for the majority of older persons with breast cancer. The study by Garg et al. in colorectal patients receiving 5-fluorouracil [31], significantly correlated shorter leukocyte TL with higher risk of hematological toxicities and mucositis. With regard to BC, to our knowledge only 2 rather small-scale studies have been carried out to analyze a potential relationship between TL and chemotherapy-induced toxicities. In the first study [18], conducted by our own group, TL also did not predict chemotherapy toxicity in 56 older breast cancer patients receiving adjuvant docetaxel and cyclophosphamide. In a second study by Quintela-Fandino et al. [34], TL was measured in blood samples from 115 treatment naive patients from a clinical trial in early HER2-negative BC. It reported that the load of critically short telomeres, rather than low average TL, predicted an almost two-fold incidence of chemotherapy-related side effects. Our results cannot be compared to the Quintela-Fandino study for several reasons. Firstly, only one BC subtype was included here, while our cohort comprised all BC subtypes. Secondly, we only measured average TL and not individual TL, so that critically short telomeres cannot be identified in our study. Thirdly, in the other study, patients received paclitaxel instead of FEC. With regard to the treatment regimen, it should also be noted that fluorouracil was routinely added to anthracycline and cyclophosphamide treatment during the study period (2007–2010). However, a study presented in 2013 [35] showed that fluorouracil did not improve outcome while increasing side effects. This study led to the definitive omission of fluorouracil from (neo)adjuvant anthracycline regimens. It is not known if the abolition of fluorouracil would have impacted telomere length differently, but this seems rather unlikely.

Our results show no significant correlation between TL measured at diagnosis and patient survival outcomes (neither OS, BCSS nor DDFS) in BC patients receiving FEC or FEC-D. TL has already been shown to be a predictor of mortality in other diseases such as cardiovascular disease [36] and various cancer types. Previous studies have significantly associated shorter TL with poorer prognosis in colorectal, prostate, bladder, ovarian, lung cancer and leukemia [33,[37], [38], [39], [40], [41]]. Conversely, other studies have reported an inverse correlation, suggesting that shorter TL would indicate better outcome in some cancers such as kidney or liver cancer [42,43]. In BC, previous studies have similarly reported conflicting evidence. Some of them suggest that shorter TL significantly correlates with poorer prognosis in BC patients [44], [45], [46], while others state that the opposite is true [47], [48], [49]. Besides, some studies propose that the relationship between TL and BC is more complex, and that a significant correlation can only be observed in some specific settings. For instance, a case-control study suggested that short TL is indicative of poorer survival only in patients with advanced stage [48], while another paper reported that longer TL was only significantly associated with worse outcome in HER2 negative cases [49]. In our study, we did not find any significant association between TL and outcome in 445 BC patients, and this did not change after correction for risk factors like stage and subtype (see Table 5), so our data add to the evidence of a limited impact of TL on outcome. Discrepant results observed in previous studies may be due to differences in composition and size of the study populations, the influence of confounding factors or the design of the study. For example, only a part of the included BC patients had received chemotherapy in some studies, which is different in our study. Moreover, DNA samples were obtained at different times during the disease course in the different studies, what could also make the results incomparable, since cancer itself and/or cancer treatment could cause changes in TL. Furthermore, inconsistent results may be due to differences in the measurement techniques, although a good correlation between the Southern blot ‘telomere restriction fragment (TRF)’ method and the qPCR-based method has been reported. Another possible explanation for the discordance is the variation in the origin tissue of the DNA. A systematic review of 36 studies analyzing the relationship between TL and BC prognosis showed an overall trend towards a positive association of longer TL and better prognosis in those studies analyzing telomeres from tumor samples, but not in those analyzing peripheral blood leukocyte TL [50]. Despite the fact that it has been proven that peripheral blood leukocyte TL reliably represent other tissues’ TL [51], carcinogenic processes might disrupt this relationship, this being a possible explanation to our negative results. Even so, prognostic value of TL could be based on other mechanisms independent of tumor features. Short telomeres can induce senescence of cells, including immune cells. Accordingly, one study proposed that short telomeres would be indicative of a lower defense capacity of the organism and, therefore, of a worse prognosis [37]. Another report suggests that TL changes throughout cancer progression, either increasing or diminishing, and that it is the extent of shortening, rather than short TL at baseline, that actually correlates with worse outcome [52]. In order to confirm this theory, further studies must be done in which TL is measured at different moments of the disease. Unfortunately, our blood collection does not contain longitudinal samples, so new prospective studies with newly diagnosed BC patients would be required. Finally, it is important to note that age-related processes, like ‘inflammaging’, may cause a non-linear course of TL shortening during life. Consequently, longitudinal TL analyses in one individual may reveal an acceleration of TL shortening at higher age. A subanalysis on the older population (e.g. ≥60 years) could therefore be interesting, but our breast cancer cohort is relatively "young" and the older subgroup is too small to study this effect.

The main strength of our study is the use of a large single center cohort with a long follow-up reporting detailed survival outcomes and acute as well as long-term side effects. In addition, our database also included many other factors related to patients, tumor features and treatment regimens, which allowed us to correct for potential confounding factors. Moreover, patients in our cohort are representative of the population being diagnosed with BC and receiving (neo-)adjuvant chemotherapy, and they were treated homogeneously with FEC or FEC-D. Also, we used a well-established and reliable TL measurement technique. Lastly, our results demonstrated an inverse correlation between age and TL that has already been well-stablished in previous studies [53,54], while no other correlations were found. These results prove that the protocols were performed accurately, and therefore, they support the reliability of our results.

There are also some limitations to this study. Except for neutropenia related events, the number of other severe side effects was rather low and for some very low (e.g. grade 3 bleeding, grade 3–4 thrombocytopenia, cardiac failure), thus not allowing solid conclusions. Moreover, we only measured TL in peripheral blood leukocytes, while it would have been interesting to measure it also in tumoral tissue and in normal tissue adjacent to the tumor. Thirdly, although two different groups were determined according to the treatment (FEC and FEC-D), drug-specific toxicity cannot be discriminated between the individual chemo components (fluorouracil, epirubicin, cyclophosphamide or Docetaxel). In this regard, another limitation of our study is that no information was available on dihydropyrimidine dehydrogenase (DPYD) mutational status, as it was not routinely assessed during the study period. DPYD mutations occur in about 8% of humans (about 7% heterozygous mutation with moderate impact on fluorouracil toxicity, and about 1% homozygous mutation with major impact on fluorouracil metabolism and toxicity). It is not known whether DPYD mutations would influence the impact of fluorouracil on telomere length. Finally, (neo-)adjuvant anthracyclines are given more and more in a dose dense fashion with primary G-CSF prophylaxis. Neutropenic events will anyhow become lower if G-CSF is used prophylactically, but on the other hand the chemotherapy dose intensity is higher which may also impact the chemotherapy impact on TL.

The main conclusion of the present study is that in BC patients treated with FEC or FEC-D there is no significant correlation between baseline TL and chemotherapy-related toxicities nor with long term outcome. Nevertheless, further investigations should be carried out in order to validate these findings in other BC chemotherapy regimens and settings. The identification of new prognostic and predictive biomarkers would clearly facilitate treatment individualization, leading to better outcome and quality of life for the patients, especially in the elderly.

The other authors declare no conflict of interest.

## CRediT authorship contribution statement

**Sigrid Hatse:** Conceptualization, Methodology, Writing – original draft, Supervision. **Marta Serena:** Conceptualization, Methodology, Writing – original draft. **Christof Vulsteke:** Methodology, Resources, Writing – review & editing. **Kevin Punie:** Methodology, Resources, Writing – review & editing. **Patrick Neven:** Methodology, Resources, Writing – review & editing. **Ann Smeets:** Methodology, Resources, Writing – review & editing. **Annouschka Laenen:** Formal analysis, Writing – review & editing. **Hans Wildiers:** Conceptualization, Methodology, Resources, Writing – original draft, Supervision.

## Declaration of Competing Interest

Hans Wildiers's institution received financial compensation on his behalf for advisory boards, lecture fees and/or consultancy fees from Immutep Pty, MSD, Astrazenca Ireland, Daiichi, AbbVie, Lilly, PSI CRO AG, KCE, EISAI, Astrazeneca, Roche. Hans Wildiers's institution received an unrestricted research grant on his behalf from Roche and he received travel support from Pfizer and Roche. Christof Vulsteke: Research funding paid to institution from Merck MSD; Consulting fees from GSK, Astellas Pharma, Merck MSD, BMS, Leo-Pharma, Janssen, Cilag, Bayer, and AstraZeneca. Kevin Punie received honoraria for consultancy/advisory roles or speaker fees from Astra Zeneca, Eli Lilly, Gilead Sciences, MSD, Novartis, Roche, Seagon (personal, outside the submitted work). Kevin Punie's Institution received honoraria for consultancy/advisory roles or speaker fees from Astra Zeneca, Eli Lilly, Gilead Sciences, Medscape, MSD, Mundi Pharma, Novartis, Pfizer, Pierre Fabre, Roche, Seagen, Teva, Vifor Pharma (paid to institution, outside the submitted work). Research funding from MSD and Sanofi (paid to institution, outside the submitted work). Travel support from Astra Zeneca, Novartis, Pfizer, PharmaMar, Roche (outside the submitted work).
